# Preparation of TiO_2_ Superhydrophobic Composite Coating and Studies on Corrosion Resistance

**DOI:** 10.3389/fchem.2022.943055

**Published:** 2022-07-08

**Authors:** Chaogang Zhou, Qiya Chen, Qinggong Chen, Huawei Yin, Shuhuan Wang, Chuanbo Hu

**Affiliations:** ^1^ College of Metallurgy and Energy, North China University of Science and Technology, Tangshan, China; ^2^ School of Environmental and Chemical Engineering, Chongqing Three Gorges University, Chongqing, China; ^3^ Department of Chemistry, Hong Kong Baptist University, Hong Kong, Hong Kong SAR, China

**Keywords:** coating, corrosion resistance, superhydrophobic, wettability, self-cleaning

## Abstract

The superhydrophobic coatings with excellent performance are prepared on the brass substrate to improve its application limitations in real production. In this article, the superhydrophobicity was obtained by the modification of TiO_2_ nanoparticles, and the FAS/STA-TiO_2_ superhydrophobic coating of the composite structure was obtained by modification of 1, 1, 2H, 2H-perfluoroquine trimethyl silane (FAS). By using scanning electron microscopes (SEMs), X-ray spectrometers (EDSs), and Fourier transform infrared (FTIR) spectrometers, the surface morphology, chemical composition, and functional group structure of the samples were analyzed in turn. Experiments show that the water contact angle of the FAS-modified STA-TiO_2_ coating reaches 161.3°, and the sliding angle is close to 1.2°. Based on the chalk dust containment, it has enabled noticeable self-cleaning properties. The composite superhydrophobic coating also presents enhanced adhesive strength compared with the single coating by the tape peeling experiment. Moreover, the composite coating has a corrosion current density as low as 8.41 × 10-7 A/cm2, and the largest |Z| in low frequency in a 3.5% NaCl solution to achieve better protection of the brass substrate. It is also not difficult to see that FAS/STA-TiO_2_ coating can not only improve the corrosion resistance of brass substrates but also be applied to other metal substrates.

## Introduction

With the country's industrial development entering the middle and late times, the shortcomings of narrow application scope and insufficient support of basic materials are increasingly revealed when facing the high-quality development requirements. Therefore, promoting the functional transformation of basic materials and broadening the scope of application have become the top priorities of current industrial development. Brass has the advantages of good electrical conductivity, thermal conductivity, wear resistance, good strength, and toughness and is widely used in machinery manufacturing, national defense industry, instrumentation, and other fields (Li et al., 2021; Zheng et al., 2021). Found in industrial research, brass is susceptible to stains, pressure, corrosive media, and so on. More and more people can pay attention to durability and anti-corrosion. Comparing the common protected procedures, superhydrophobic coating has a good performance of self-cleaning (Xu et al., 2017; Wang X et al., 2021), antifouling and deicing (Zhang et al., 2017; Zhang et al., 2021; Li et al., 2022), sliding drag reduction (Pakzad et al., 2020), corrosion resistance (Xu et al., 2011; Chen et al., 2021), oil-water separation (Liao et al., 2021; Lv and Lin, 2021; Suk et al., 2021), and so on. Xiang et al. (Xiang et al., 2022) designed a stable slippery surface which consists of compact-nickel-underlayer/porous-nickel-midlayer/PDMS/paraffin-infused top layer and mainly applied for corrosion protection. This special triple-layered structure can broaden the application of marine and other fields. Roshan et al. (Roshan et al., 2022) provided a two-component coating using fluoropolyurethane and varying concentrations of surface-modified silica nanoparticles. They reported that the effective anticorrosion resistance of the superhydrophobic coating was about 25 times greater than that of the blank sample.

Superhydrophobic coating refers to coatings whose surface has a water contact angle (WCA) greater than 150° and a rolling angle (SA) less than 10° (Zhang et al., 2020). The superhydrophobicity of building coating materials is mainly divided into two aspects: one is micro-nano materials with rough structure (Xu and Li, 2021), and the other is modifiers with low surface energy (Lu et al., 2009; Hu et al., 2021). Wang et al. (Wang C et al., 2021) prepared TiO_2_ microspheres that integrate the micro-nanostructure, mixed with epoxy resin, and spread onto the glass slide to obtain a superhydrophobic coating. This kind of coating can put up with 100 times of tape peeling and have good stable rough performance. Zheng et al. (Zheng et al., 2022) used a spraying method to obtain a SiO_2_/HDTMS-ZnO/PPS superhydrophobic composite coating. In view of its good hydrophobicity, it has excellent advantages in antifouling, heat stability, and corrosion resistance. Common preparation methods include chemical etching, sol-gel, electrochemical deposition, layer-by-layer self-assembly, and so on. Among them, most of the methods are difficult to mass-produce in actual production due to factors such as poor adaptability, expensive instruments, and complicated process operations (Li et al., 2019; Li et al., 2020; Wan et al., 2021). In comparison, the dip-coating method has strong practicability, low cost, and a simple process. It is a more commonly used method for preparing superhydrophobic coatings.

In this article, a simple dip-coating method was used to prepare superhydrophobic TiO_2_ coatings on the brass substrate. Stearic acid (STA) was used as a low surface energy substance to modify TiO_2_ nanoparticles to prepare STA-TiO_2_ superhydrophobic coating solution, and then a certain amount of 1, 1, 2H, 2H-perfluoro-decyltrimethoxysilane (FAS) to obtain a FAS/STA-TiO_2_ composite coating with a superhydrophobic structure, reducing the adverse effects of the brass matrix material in the environment. The resulting composite surfaces are characterized by the surface morphology, chemical composition, and functional group structure. Furthermore, we explored the variations in hydrophobic properties of single and composite coatings through wettability, self-cleaning, adhesion, and corrosion resistance (Xie et al., 2018; Ali et al., 2020).

## Experimental Section

### Materials

Stearic acid (STA), sodium dodecylbenzenesulfonate (SDBS), anhydrous ethanol, and acetone were purchased from Chengdu Kelong Chemical Co., Ltd. TiO_2_ nanoparticles (40 nm, anatase type) were purchased from Shanghai Chaowei Nanotechnology Co., Ltd. 1, 1, 2H, 2H-perfluoro-decyltrimethoxysilane (FAS, 98%) was purchased from Guangdong Wengjiang Chemical Reagent Co., Ltd. Y-aminopropyltriethoxysilane (KH550) was obtained from Shandong Yousuo Chemical Technology Co., Ltd. Deionized water was made in the laboratory.

### Preparation of STA-TiO_2_ Powders

The modified TiO_2_ particles were prepared by the hydrothermal method. First, 5 g of TiO_2_ was added to 125 ml of ethanol solution and stirred at room temperature for 2 h; then 1 g was added to 125 ml of ethanol solution and stirred in a constant temperature water tank at 40°C for 2 h. Finally, the homogeneous TiO_2_-ethanol solution and the STA-ethanol solution were mixed and stirred at a constant temperature of 70°C for 8 h to obtain a white mixed suspension. After cooling at room temperature, the suspension was centrifuged, dried in a constant temperature drying oven at 60°C for 5 h, and ground to obtain a white modified TiO_2_ powder (STA-TiO_2_).

### Preparation of Coating Liquid

The obtained STA-TiO_2_ powder was dissolved in ethanol solution, sonicated at room temperature for 20 min, followed by adding a certain amount of curing agent (KH550) and dispersing agent (SDBS); then stirred at room temperature for 2 h to form a uniform white coating liquid STA-TiO_2_. Under the same conditions, the FAS-ethanol solution prepared in the ratio of 1:10 was added to the STA-TiO_2_ coating solution to obtain the FAS/STA-TiO_2_ coating solution.

### Preparation of the Superhydrophobic Surface

**FIGURE 1:**
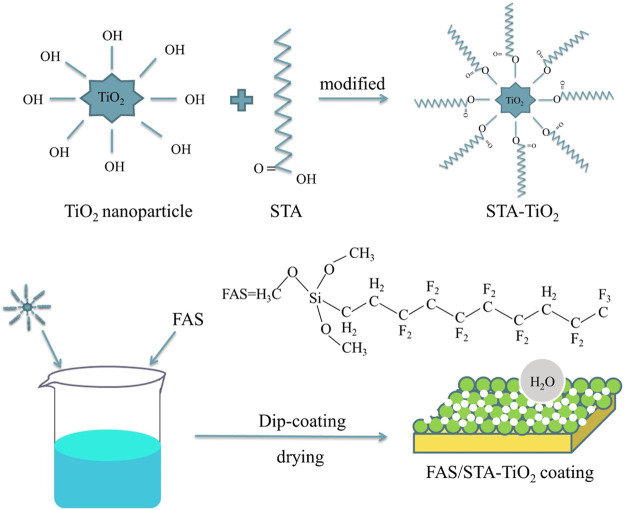
Schematic diagram of preparing FAS/STA-TiO_2_ composite coating on the brass surface.

The brass sheets with specifications of 20 mm × 20 mm and 20 mm × 40 mm were polished with 180, 400, 600, 1,000, and 1,200 grit metallographic sandpapers, respectively, until the surface was smooth and without obvious scratches. Then, ultrasonic cleaning was performed in acetone, absolute ethanol, and deionized water for 5 min in turn. The previously treated brass sheets were immersed in the ethanol solutions of FAS, STA-TiO_2,_ and FAS/STA-TiO_2_, respectively, and let stand for 20 min until a uniform film was formed on the brass surface. Finally, the film-formed brass sheet was dried at room temperature for 5 min and then dried in a constant temperature drying oven at 70°C for 30 min to obtain FAS coating, STA-TiO_2_ coating, and FAS/STA-TiO_2_ coating. Figure 1 is a schematic diagram of the preparation of the FAS/STA-TiO_2_ composite coating on the brass surface.

### Characterization

The static contact angle of the sample (20 mm × 20 mm) was measured using a Dataphysics OCA20 contact angle meter at room temperature. The volume of deionized water used was 3 μL, and the contact angle value was the average of five measurements (five different points). The measurement results are based on the measurement average. Ultra Plus field emission scanning electron microscope (SEM) was used for scanning, and the microscopic morphology and elemental composition of the coating surface were observed with the energy dispersive spectrometer (EDS); Fourier transform infrared spectroscopy (FTIR) was used to detect the coating surface. The chemical functional groups of the test are in the range of 4,000–500 cm^−1^. The corrosion resistance of the samples was tested by a CHI660E electrochemical workstation. The scan rate was 2 mV/s, the stabilization time was 2 s, and the sensitivity was 10^−6^ A/V. The electrochemical test used a three-electrode system, with a sample of size 20 mm × 40 mm as the working electrode, a platinum electrode (Pt) as the counter electrode, and a saturated calomel electrode (SCE) as the reference electrode. The Tafel polarization curves of the samples were tested in the range of -500–500 mV. Electrochemical impedance spectroscopy (EIS) plots were generated using a 10 mV sinusoidal disturbance and a test frequency range of 100 k–0.01 Hz at open circuit potential.

## Results and Discussion

### Analysis of the STA-TiO_2_ Nanoparticle Structure

**FIGURE 2:**
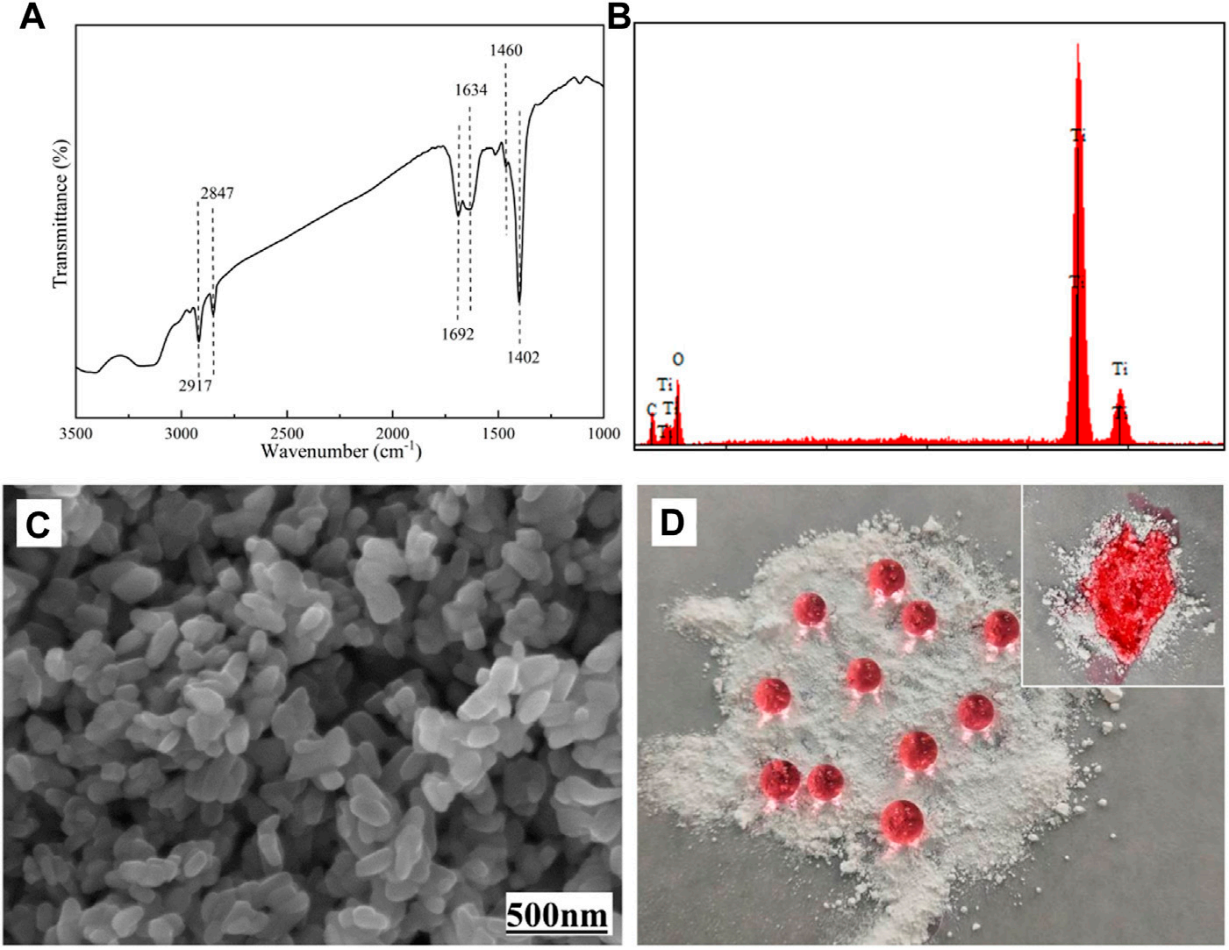
Characterization of STA-TiO_2_ powders. **(A)** FTIR spectrum, **(B)** EDS spectrum, **(C)** SEM image, and **(D)** optical images.

As shown in Figure 2, the structure of STA-TiO_2_ powder was characterized by FTIR, EDS, SEM, and optical image. From the FTIR spectrum of Figure 2A, it is found that the sharp peaks at 2,847 cm^−1^ and 2,917 cm^−1^ correspond to the C-H stretching vibrations in methylene and methyl groups, respectively. The sharp peak at 1,460 cm^−1^ is mainly sheer vibration of the methylene group or the symmetrical deformation vibration of the methyl group; the bending vibration absorption peak of O-H at 1,402 cm^−1^ indicates that there is a long carbon chain formed by the combination of stearic acid and TiO_2_ nanoparticles in the product. At the same time, the characteristic peaks of C=O appeared at 1,634 cm^−1^ and 1,692 cm^−1^ on the TiO_2_ nanoparticles modified with stearic acid, indicating that stearic acid and TiO_2_ nanoparticles were combined in a bidentate coordination manner. In Figure 2B of EDS component analysis, except with Ti and O elements, a C element can be found, indicating that stearic acid has successfully modified the TiO_2_ nanoparticles. Figure 2C shows the surface morphology of the STA-TiO_2_ powder under the scanning electron microscope. It can be seen that the particle size of the modified TiO_2_ is reduced, the particle distribution is relatively uniform, and the agglomeration is significantly weakened. Figure 2D shows the colored water droplets are placed on the TiO_2_ particles and the STA-TiO_2_ particles, respectively. Due to the low surface energy of STA, the water droplets present an independent circular shape on the STA-TiO_2_ particles, which proves that STA-TiO_2_ particles have superhydrophobic properties.

### Chemical Composition

**FIGURE 3:**
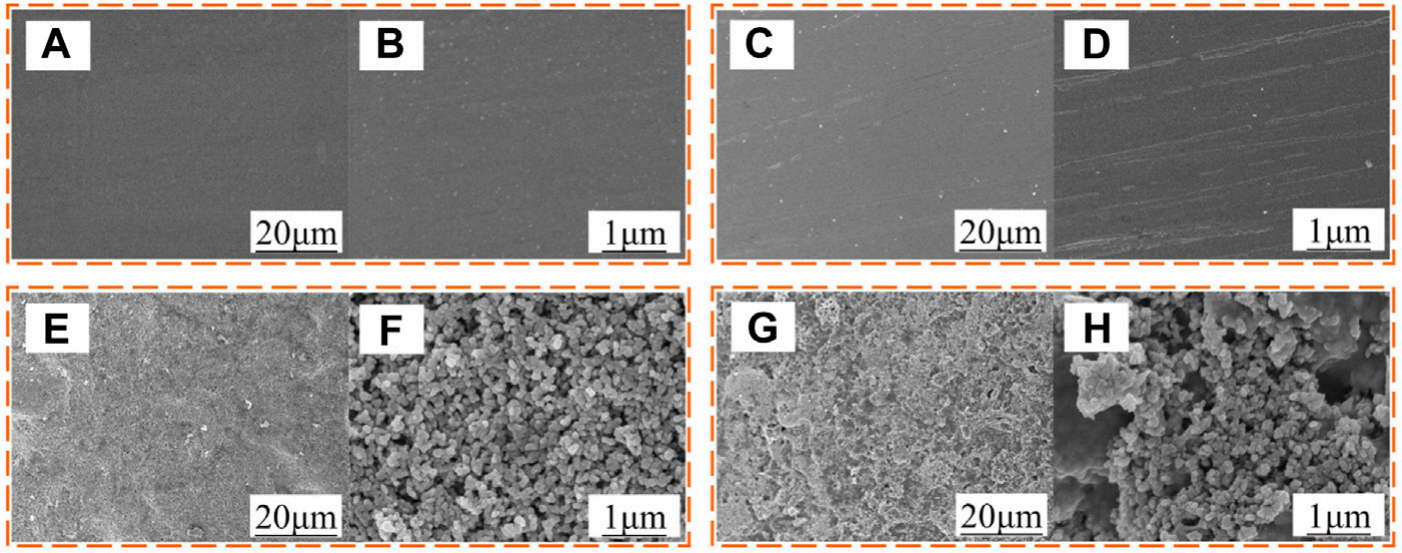
SEM images of the samples. **(A,B)** Brass, **(C,D)** FAS coating, **(E,F)** STA-TiO_2_ coating, and **(G,H)** FAS/STA-TiO_2_ coating.

Figure 3 shows the surface morphologies of brass, FAS, STA-TiO_2,_ and FAS/STA-TiO_2_ coatings. As shown in Figures 3A and B, it can be seen that the brass surface is flat and smooth and the contact area with water droplets is large. As shown in Figures 3C and D, when using FAS-modified brass, its contact angle reaches 115.2°, which plays a role in isolating the contact between the air and the substrate surface. Figure 3E is the image of the sample at low magnification, and the surface morphology is blurred; while in the image at high magnification (Figure 3F), the nanoparticles show prominent nanostructures stacked between them. Figures 3E and F show that the TiO_2_ particle size on the surface of the stearic acid-modified coating is uniform. There are voids between the particles, the surface distribution is relatively rough, and a clear micro-nanostructure is formed. This structure can capture a large amount of air to form an “air cushion” structure between the water droplets and the substrate and reduce the solid-liquid contact area, which conforms to the structural equation of t/he heterogeneous rough surface proposed by Cassie, and the contact angle reaches 155°. As shown in Figures 3G and H of the coating after FAS composite, the TiO_2_ particles are evenly distributed on the surface of the coating formed by FAS. The surface features are more diverse, and the pores are increased. The adhesion between the coating molecules is improved, and the surface has a clear bonding part—low surface modification bonding. The obtained coating has a smaller contact area with water droplets; the contact angle value reaches 161.3° and has more excellent superhydrophobicity.

**FIGURE 4:**
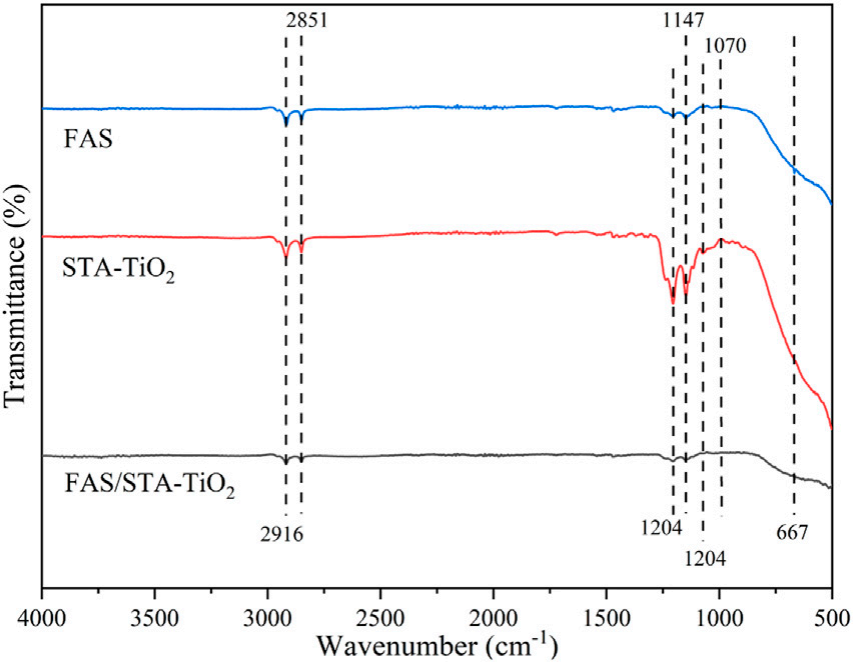
FTIR spectra of FAS, STA-TiO_2_, and FAS/STA-TiO_2_.

To investigate the chemical compositions of FAS, STA-TiO_2,_ and FAS/STA-TiO_2_ coatings, FTIR spectra and EDS were recorded and analyzed. Comparing the spectral lines of the three coatings (Figure 4), it can be seen that similar characteristic peaks appear at the wavenumbers of 2,916 cm^−1^ and 2,851 cm^−1^, which are similar to those of KH550. The peaks correspond, demonstrating the presence of KH550 on the coating surface. The characteristic absorption peak of the FAS/STA-TiO_2_ coating at the wavenumber of 1,070 cm^−1^ is generated by the Si-O-Ti bond stretching vibration, which is mainly caused by the dehydration condensation reaction between the hydrolyzed FAS and the hydroxyl group on the surface of TiO_2_. At the same time, at the wavenumbers of 1,204, 1,147, and 1,114 cm^−1^ for the STA-TiO_2_ coating, the peaks correspond to the modified TiO_2_ fluctuated greatly. After adding FAS to the STA-TiO_2_ coating, the F content increased and the peak value of this band increased. Therefore, the weakening of the peak intensity is mainly due to the broad characteristic absorption peaks of TiO_2_ particles. The C-F bond of methylene and methyl groups in FAS is weakened after recombination, that is, FAS is successfully grafted to the surface of TiO_2_. Comparing the element contents of the three samples (Table 1), it can be clearly found that the samples coated with STA-TiO_2_ and FAS/STA-TiO_2_ have a significantly lower content of Cu element than the FAS coating, which proves that the composite coating has better dispersion and more comprehensive coverage.

**TABLE 1:** Element contents of various films.

Element	C/%	N/%	O/%	F/%	Si/%	Cu/%	Ti/%
FAS	1.2	0.765	1.188	0.739	0.601	95.508	—
STA-TiO_2_	2.36	0.091	12.102	0.859	1.017	4.556	79.015
FAS/STA-TiO_2_	1.862	0.969	17.39	3.905	1.431	3.107	71.335

**FIGURE 5:**
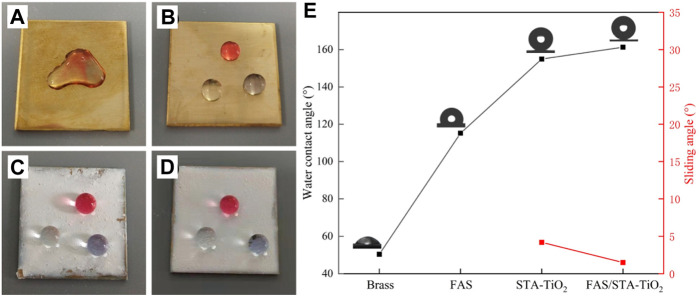
Photographs of droplets on different coatings. **(A)** Brass, **(B)** FAS, **(C)** STA-TiO_2_, and **(D)** FAS/STA-TiO_2_. **(E)** Changes in the contact angles of different coatings.

Optical images of droplets placed on different coating surfaces are shown in Figure 5. Figure 5A shows the wetting of the brass surface. It can be found that the water droplets of the three colors collapsed on the surface of the substrate and converged together, showing obvious hydrophilicity. In Figure 5B of the brass substrate after FAS modification, the water droplets are slightly protruding and do not aggregate together, proving that the low surface modifier FAS indeed reduces the surface energy of brass. Figures 5C and D show the surface wetting of STA-TiO_2_ and FAS/STA-TiO_2_ coatings. The water droplets present a more stretched spherical structure, which can be removed from the surface without a slight touch of external force. Among them (Figure 5E), the STA-TiO_2_ coating has a contact angle of 155° and a rolling angle of 3.6°; the FAS/STA-TiO_2_ coating has a contact angle of 161.3° and a rolling angle of up to 1.2°. Through observation and analysis, after the construction with micro-nano rough structure and low surface modification, the contact area between the coating surface and the water droplet is reduced, which is in accordance with the Cassie–Baxter model structure.

### Self-Cleaning Ability

**FIGURE 6:**
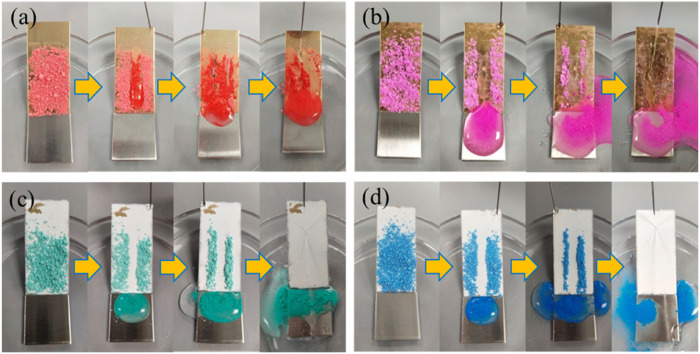
Self-cleaning properties of different coating surfaces. **(A)** Brass; **(B)** FAS; **(C)** STA-TiO_2_; and **(D)** FAS/STA-TiO_2_.

Self-cleaning is the basis for the application of superhydrophobic coatings. The coating was placed at an inclination angle of less than 10°, and the surface was covered with chalk dust as a contaminant. From Figure 6A, it can be found that the water droplets adhered and slide down along the powder on the brass surface, leaving a lot of stains on the sample. While the brass is modified by FAS (Figure 6B), the surface composition is a low structure that isolates the contact between the outside and the substrate but does not constitute a superhydrophobic structure.

Based on the “air cushion” model proposed by Cassie, the surface wetted area with a rough structure and low surface chemical composition is smaller than that of the copper alloy surface. When the surface is inclined at a certain angle, the water droplets on the superhydrophobic surface with the composite structure are very easy to roll and take away a large amount of powder, leaving a clean and dry channel without powder and showing good self-cleaning properties. As shown in Figures 6C,D, when the water droplets land on the superhydrophobic STA-TiO_2_ coating and the FAS/STA-TiO_2_ coating, the water droplets are mixed with chalk ash on the inclined surface to form a spherical sludge that rolls down rapidly without stain residue. Among these, the water droplets on the surface of the FAS/STA-TiO_2_ coating had enhanced bounciness and stronger decontamination ability than the STA-TiO_2_ coating. Hence, it can be concluded that the superhydrophobic FAS/STA-TiO_2_ coating has a good self-cleaning ability and can be applied in real life.

### Mechanical Stability

**FIGURE 7:**
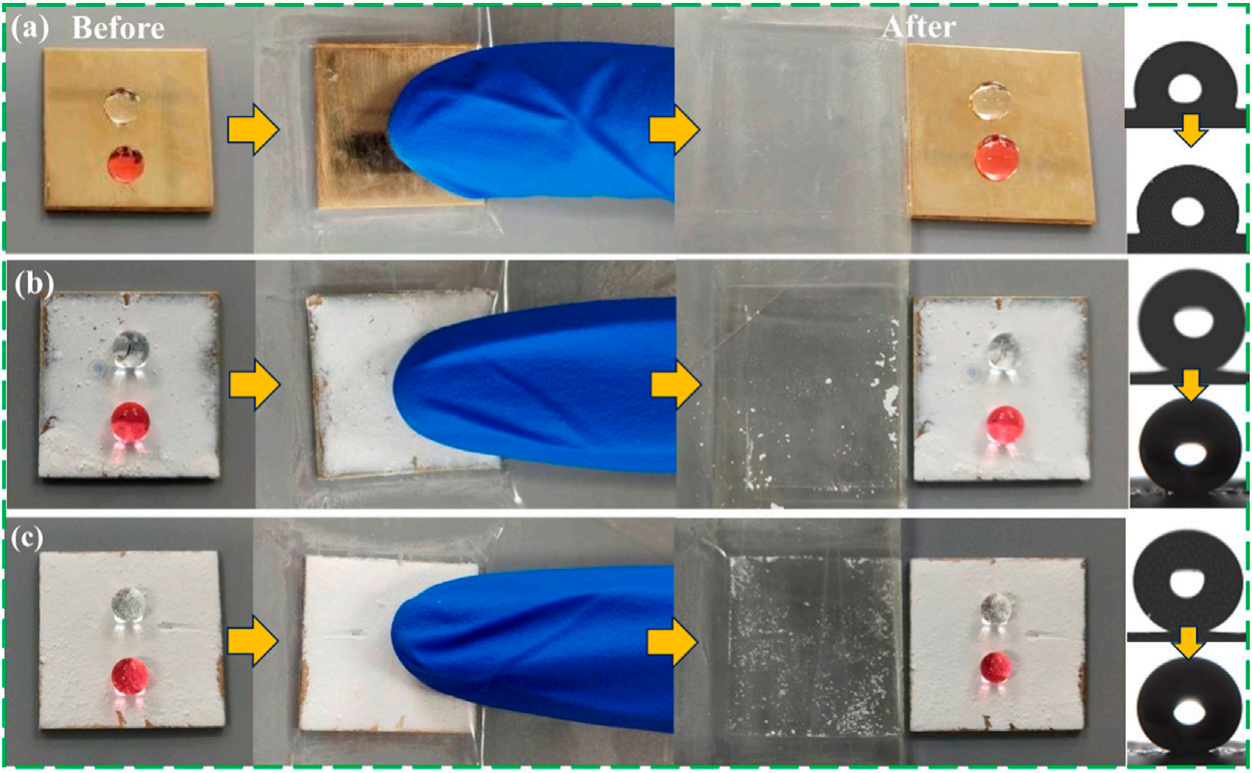
Adhesion results of superhydrophobic coatings. **(A)** FAS, **(B)** STA-TiO_2_, and **(C)** FAS/STA-TiO_2_.

The superhydrophobic coating is prepared by the dip-coating method, and the adhesion between the coating material and the substrate is easily damaged by external factors, which reduces the superhydrophobicity of the surface. In order to detect the performance change of the coating material under certain mechanical pressure and wear conditions, as shown in Figures 7A–C, the tape peeling experiment was used to simulate the external pressure environment to test the coating adhesion. This experiment needs to adhere the coated surface to the surface of the 3M VHB strong tape and press repeatedly on the sample of the tape with the finger, then pull the tape to test its adhesion after 20 times. By observing the contact angle of water droplets on the surface of the material, it can be found that under the combined action of pressure and adhesive tape, the superhydrophobicity of the material decreases. The contact angle of the FAS/STA-TiO_2_ coating still reaches 159.8°, and the water droplets are round and spherical on the coating surface. At the same time, comparing the samples before (Figure 7B) and after adding FAS (Figure 7C), it is found that the fluorinated structure formed by FAS and STA-TiO_2_ is stable and can well resist external mechanical damage.

### Anti-Corrosion Performance

**FIGURE 8:**
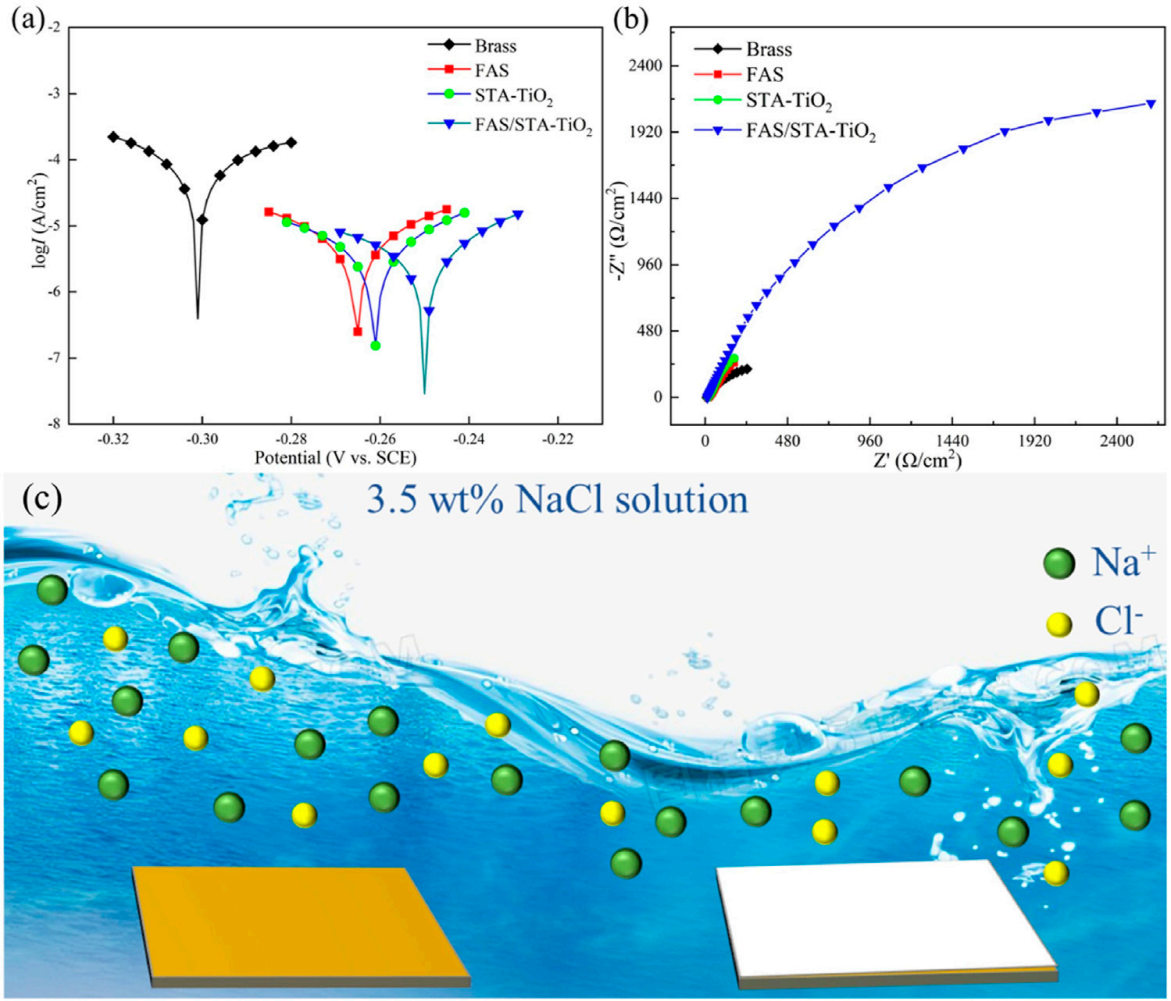
**(A)** Tafel plots, **(B)** Nyquist plots, and **(C)** anti-corrosion mechanism of samples soaked in 3.5 wt% NaCl solution for 2 h, respectively.

The anti-corrosion performances of the untreated brass samples and those coated with FAS, STA-TiO_2,_ and FAS/STA-TiO_2_ were tested by an electrochemical workstation in the electrolyte of NaCl solution with a mass fraction of 3.5 wt%. The potentiodynamic polarization curves and Nyquist plots are shown in Figures 8A and B. The corrosion potential (*E*_coor_) and corrosion current (*I*_coor_) were fitted by the Tafel linear extrapolation method, and the fitted corrosion parameters are shown in Table 2.

**TABLE 2:** Corrosion parameters of different samples soaked in 3.5 wt% NaCl solution for 2 h were fitted by Tafel curves.

Sample	*E*_coor_ (V)	*I*_coor_ (A/Cm^2^)	η/%
Brass	−0.30	4.86 × 10^−6^	—
FAS	−0.264	2.81 × 10^−6^	42.18
STA-TiO_2_	−0.261	1.62 × 10^−6^	66.67
FAS/STA-TiO_2_	−0.25	8.41 × 10^−7^	82.70

It can be found from Table 2 that the bare brass has a lower *E*_coor_ and a higher *I*_coor_, indicating that the bare brass sheet is easily corroded by the medium. The *E*_coor_ of the FAS modified is 36mV higher than the previous one, indicating that the surface modification of low surface substances acted as a barrier to the substrate and protected the substrate from being corroded by the medium. At the same time, for the TiO_2_ coating modified by stearic acid, the *E*_coor_ of the superhydrophobic coating is moving to -0.261 V, the *I*_coor_ is reduced to 1.62 × 10^−6^ A/cm^2^, and the corrosion protection efficiency reaches 66.67%. After FAS modification, the corrosion current density of STA-TiO_2_ coating decreased by an order of magnitude, reaching ×8.4110^−7^A/cm^2^. Compared with the bare FAS coating and the STA-TiO_2_ coating, the FAS/STA-TiO_2_ coating provides both roughness and composite low surface energy, traps a certain amount of air, and provides a solid-air-liquid structure to prevent water droplets from intervening. Therefore, the coating structure formed on the surface of the sample is more compact.

EIS experiment was further used to evaluate the electrochemical behavior of these samples. It can be seen that the FAS/STA-TiO_2_ exhibited the largest semicircle, obviously, while the brass substructure displayed a small one. This result further confirmed that the FAS/STA-TiO_2_ coating exhibited the best anti-corrosion performance. As we all know, the common equivalent circuit model of the samples in the immersion environment is two-time constants. As shown in Figure 8B, all of the plots have a small semicircle in the high-frequency range or a large semicircle in low-frequency range. Also, the |Z| in high frequency was connected with the surface of samples while low frequency represents the impedance of the barrier layer (Xiang et al., 2021). The composite coating showed the largest |Z| in low frequency which revealed a large impedance of the barrier layer and demonstrated good anti-corrosion performance. The anti-corrosion mechanism of samples was described in Figure 8C. The composite superhydrophobic coating can provide more excellent anti-corrosion performance and weaken penetration of the interface. In the meantime, the FAS-modified STA-TiO_2_ coating has a smoother surface and effectively protects the substrate.

## Conclusion

In summary, nanoparticles with superhydrophobic properties were prepared by hydrothermal method with TiO_2_ as the rough structure and stearic acid (STA) as a low surface energy modifier. Then fluorinated with 1, 1, 2H, 2H-perfluorodecyltrimethoxysilane (FAS) to construct a FAS/STA-TiO_2_ superhydrophobic composite coating with a contact angle of 161.3° and a rolling angle of 1.2°. Aiming at the disadvantage of poor durability of coating materials, silane coupling agent KH550 and 1, 1, 2H, 2H-perfluorodecyltrimethoxysilane (FAS) were used, which can be used as adhesives, reinforcing materials, and substrates. The combination can also form a film on the surface of the material to improve the hydrophobicity. The obtained FAS/STA-TiO_2_ composite coating has protection efficiency in 3.5wt% NaCl solution, and its corrosion resistance is stronger than that of a single coating. Moreover, it has a good application prospect in actual production and life.

## Data Availability

The original contributions presented in the study are included in the article/Supplementary Material; further inquiries can be directed to the corresponding author.
